# Microstructure and Mechanical Properties of As-Built Ti-6Al-4V and Ti-6Al-7Nb Alloys Produced by Selective Laser Melting Technology

**DOI:** 10.3390/ma17184604

**Published:** 2024-09-19

**Authors:** Dorota Laskowska, Błażej Bałasz, Wojciech Zawadka

**Affiliations:** Faculty of Mechanical Engineering and Energy, Koszalin University of Technology, Śniadeckich 2, 75-453 Koszalin, Poland; blazej.balasz@tu.koszalin.pl (B.B.); wojciech.zawadka@tu.koszalin.pl (W.Z.)

**Keywords:** additive manufacturing, selective laser melting, Ti-6Al-4V, Ti-6Al-7Nb, relative density, microstructure, mechanical properties, biomedical applications

## Abstract

Additive manufacturing from metal powders using selective laser melting technology is gaining increasing interest in various industries. The purpose of this study was to determine the effect of changes in process parameter values on the relative density, microstructure and mechanical properties of Ti-6Al-4V and Ti-6Al-7Nb alloy samples. The experiment was conducted in response to a noticeable gap in the research on the manufacturability of the Ti-6Al-7Nb alloy in SLM technology. This topic is significant given the growing interest in this alloy for biomedical applications. The results of this study indicate that by properly selecting the volumetric energy density (VED), the relative density of the material produced and the surface roughness of the components can be effectively influenced. Microstructural analyses revealed similar patterns in both alloys manufactured under similar conditions, characterized by columnar β phase grains with needle-like α’ phases. Increasing the VED increased the tensile strength of the fabricated Ti-6Al-4V alloy components, while the opposite effect was observed for components fabricated from Ti-6Al-7Nb alloy. At the same time, Ti-6Al-7Nb alloy parts featured higher elongation values, which is desirable from the perspective of biomedical applications.

## 1. Introduction

High strength-to-weight ratios characterize titanium and its alloys for a vast range of operating temperature variations and high corrosion resistance in many chemical environments [1,2,3]. These properties create many opportunities for applications of components manufactured from titanium-based alloys, including aerospace, automotive, chemical and, most importantly, biomedical engineering. Ti-6Al-4V is the most widely used alloy for long-lasting and load-bearing bone implants in biomedical applications. Many studies highlight its high biocompatibility, although this is increasingly questioned due to the presence of vanadium [4,5]. To solve this issue, vanadium-free titanium alloys, like Ti-6Al-7Nb, were developed.

Titanium and its alloys belong to a group of materials defined as hard to machine. The challenges associated with traditional machining methods, such as milling, casting, forging or rolling, increase production costs and make manufactured parts more expensive compared to those from Fe-based alloys or Co-Cr-Mo alloys. These problems have been partially solved by additive manufacturing.

The American Society for Testing Materials (ASTM) has introduced a definition of additive manufacturing (AM) as the process of joining successive layers of material (e.g., powder) based on a 3D model of production parts [6,7]. An important advantage of additive manufacturing, especially in the case of biomedical applications, is customization. AM allows for the production of medical implants tailored to specific patient requirements [8]. Although Ti alloys show a lower elastic modulus compared to other implant materials (e.g., Co-Cr-Mo), it is higher than the elastic modulus for the bone tissue being replaced, which can lead to the so-called stress-shielding phenomenon [9,10]. The characteristics of AM processes make it possible to fabricate components with complex geometry and spatial porosity (designed based on TPMS topology) and to define the directionality of mechanical properties, designing so-called architectural materials [11,12,13,14]. As studies show, this approach allows us to reduce the stiffness of the fabricated implant, thus reducing the risk of stress shielding [15].

Additive manufacturing has drawbacks that limit its application in large-scale and mass production. The most significant issues include: a reduction in mechanical properties due to internal porosity [16]; the anisotropy of mechanical properties of the produced components [17,18]; the dependence of precision and quality on various, often interrelated or mutually exclusive factors, such as the properties of the construction material or process parameters, and the necessity of post-processing to improve dimensional and shape accuracy, mechanical properties, or surface quality [19].

A widely used process for the additive manufacturing of titanium and its alloys is selective laser melting (SLM). In this technology, a laser is used as a source of thermal energy to melt and fuse a specific volume of powder [20]. The process is carried out in a protective gas atmosphere to prevent the oxidation of the molten material. The laser beam induces only part of the generation of the heat energy which is absorbed by the powder grains, while the rest of the laser beam is reflected without affecting the melting process. The local generation of thermal energy results in the formation of a molten metal pool, in which phenomena such as conduction melting [21,22], keyhole melting [23], Marangoni convection [24,25,26], alloying element segregation [19], evaporation and spattering [27] take place. The occurrence and intensity of the above-mentioned phenomena depend mainly on the volumetric energy density, but also on the conditions in the working chamber (i.e., the residual oxygen content, the temperature of the working platform, or the temperature inside the chamber). 

The state of the art on the effect of microstructure on the mechanical properties of additively manufactured Ti-4Al-6V alloy components using SLM technology is readily available thanks to the research results published in numerous publications [28,29,30,31]. However, research on the Ti-6Al-7Nb alloy has been conducted to a much lesser extent. 

Chlebus et al. [32] found that the Ti-6Al-7Nb alloy produced by SLM technology has a microstructure of columnar grains of primary β phase with long, thin α’ martensite plates. This results in higher tensile and compressive strength but lower ductility compared to the alloy produced by conventional methods. In addition, it was pointed out that the microstructure of the alloy produced by SLM technology depends not only on the process parameters but also on the spatial orientation of the manufactured object relative to the build platform. Similar conclusions were reached by a team led by Xu et al. [33], who investigated the effect of microstructure on the properties of Ti-6Al-7Nb and Ti-6Al-4V alloys. In their research, the team added a heat treatment process to the Ti-6Al-7Nb alloy. This reduced the tensile strength and hardness while increasing the elongation of the alloy. This would undoubtedly have a positive impact on the use of Ti-6Al-7Nb alloy in the production of medical implants. The referenced studies on the production of the Ti-6Al-7Nb alloy did not account for the variability in manufacturing parameters—the alloy was produced using a single strategy. From the perspective of applying SLM technology to manufacture Ti-6Al-7Nb components for biomedical applications, it seems appropriate to adopt a more comprehensive approach. 

Therefore, the purpose of this study, the results of which are presented in this article, was to determine the effect of process parameters (scanning speed and laser power) and thus the variation of the volumetric energy density on the relative density, microstructure, and mechanical properties of samples made of the Ti-6Al-4V and Ti-6Al-7Nb alloys. A comparison was made between the quality and properties of samples produced using the same manufacturing strategies for the materials under investigation. The aim was to identify the optimal (within the studied range) manufacturing strategy. The primary criterion for selecting the values of the process parameters was to maximize the relative density while minimizing the surface roughness, under the assumption that improvements in mechanical properties can be achieved by subsequent heat treatment. The described studies serve as a starting point (preliminary research) for the subsequent research stages, which will involve developing guidelines for heat treatment of the Ti-6Al-7Nb alloy to improve its mechanical properties, as well as guidelines for post-processing to reduce surface roughness.

## 2. Materials and Methods

### 2.1. Powders’ Characterization

**Figure 1:**
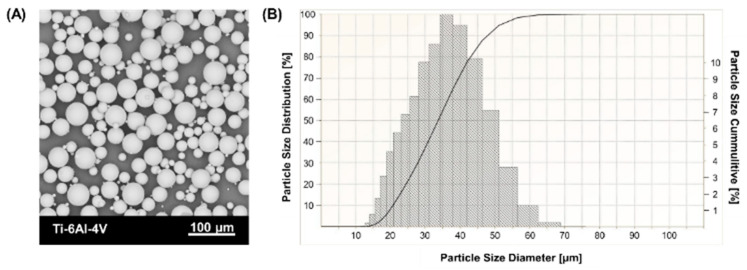
Ti-6Al-4V powder characterization: (**A**) morphology (SEM), (**B**) particle size distribution (PSD).

**Figure 2:**
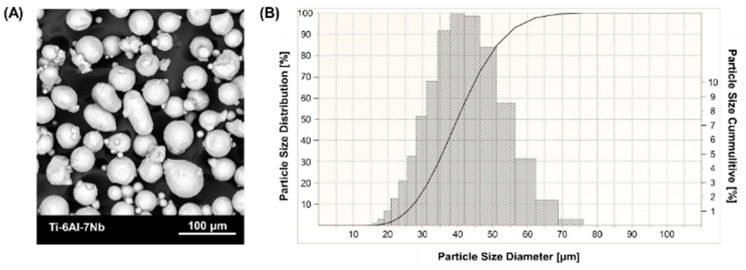
Ti-6Al-7Nb powder characterization: (**A**) morphology (SEM), (**B**) particle size distribution (PSD).

The samples were fabricated from commercial Ti-6Al-4V powder (3D Systems, Rock Hill, SC, USA) and Ti-6Al-7Nb powder (SLM Solution Group Ag, Lubeka, Germany). The chemical composition of the powders is shown in Table 1. Scanning electron microscope images (Figure 1A and Figure 2A) show spherical grain morphology with satellite characteristics for powders produced by gas atomization technology. The particle size distribution of the powder (Figure 1B and Figure 2B) was determined using an ANALYSETTE 22 MicroTec Plus laser particle size analyzer (Fritsch GmbH, Amberg, Germany) and presented according to PN-ISO 9276-1 [34].

### 2.2. Samples Fabrication

The samples were fabricated on an ORLAS CREATOR^®^ selective laser melting system (O. R. Lasertechnologie GmbH, Dieburg, Germany) with a Ytterbium fiber laser, with beam spot size 40 μm, a maximum power of 250 W and a wavelength of 1070 nm. The protective atmosphere of the working chamber was provided by the use of argon gas, which allowed the process to be performed at residual oxygen levels below 0.1%.

Volumetric energy density (E_V_) is regarded as a key metric for evaluating the complex parameters involved in selective laser melting technology, and is defined by the following equation [35,36]:

E_V_= P/(v∙h∙t), (1)

where P—laser power [W], v—scan speed [mm/s], h—hatch distance [mm], and t—layer thickness [mm]. In this work, the combination of values of individual parameters was chosen so that the volumetric energy density was in the range of 55–70 J/mm^3^. In this way, 9 manufacturing strategies were developed (Table 2).

For each manufacturing strategy, 3 cubic samples (with dimensions of 10 × 10 × 10 mm) and 5 tensile samples (dimensions defined by PN-EN ISO 6892-1:2020-05 [37]) were produced. Post-manufacturing sample preparation included mechanically removing supports and ultrasonically cleaning the samples in distilled water for 10 min.

### 2.3. Relative Density

The relative density of the manufactured samples was measured using a Mettler Toledo XS105 hydrostatic balance (Mettler Toledo, Columbus, OH, USA). Three measurements were made for each sample, resulting in nine measurements for each manufacturing strategy.

### 2.4. Surface Morphology

The surface morphology was analyzed using a PHENOM PRO scanning electron microscope (Thermo Fisher Inc., Waltham, MA, USA), with magnification in the range of 160–350,000× and a resolution of ×6 nm. Images of the top surface in the build state were captured for a randomly selected sample from each batch.

### 2.5. Surface Topography and Roughness

Surface topography was analyzed using the Olympus LEXT OLS4000 confocal microscope (Olympus, Shinjuku, Tokyo, Japan) for the upper surface of each fabricated specimen. The data acquisition area was set to 3 × 3 mm. Surface topography images were analyzed using TalyMap Platinum v7.4. software (Taylor Hobson, Leicester, UK). The data were filtered using a Gaussian filter with a length of 0.8 mm. The features of the additively manufactured surfaces were evaluated using the parameters of arithmetic mean height (Sa) and maximum height (Sz) (according to ISO 25178-2:2021 [38]), which are the most frequently used when assessing this type of surface [39,40].

### 2.6. Microstructure and Structural Defects

The structural defects analysis was conducted using the optical microscope NIKON MA200 (Nikon, Minato, Tokyo, Japan) on resin-embedded vertical cross-sections of randomly selected samples from each series. The samples were cut using a water-cooled diamond blade to prevent the sample’s overheating. The obtained cross-sections were embedded in epoxy resin DuroFast (Struers, Copenhagen, Denmark), giving them a shape suitable for further preparation using the LaboPol-30 grinder–polisher equipped with a semi-automatic LaboForce-100 head (Struers, Copenhagen, Denmark). The preparation was carried out according to the recommendations [41]. The evaluation of the grinding and polishing process was conducted using the optical microscope NIKON MA200 (Nikon, Minato, Tokyo, Japan). The metallographic specimens were cleaned using automatic cleaning Lavamin (Struers, Copenhagen, Denmark). 

The microstructure characterization was carried out on etched surfaces of metallographic specimens using the optical microscope NIKON MA200. Etching was performed for 15 s with the Kroll’s reagent (Chempur, Piekary Slaskie, Poland). 

The identification of phases was performed on metallographic specimens using an Empyrean X-ray diffractometer (Malvern Panalytical Ltd., Malvern, UK) with Cu-Kα (λ = 1.5406 Å) source. The study was conducted using Bragg–Brentano geometry within the 2θ angle range of 30–100°.

### 2.7. Microhardness Tests

**Figure 3:**
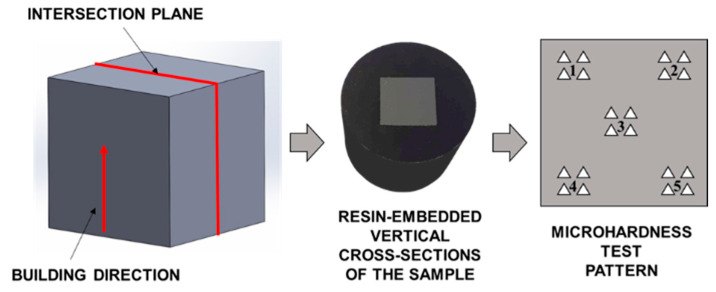
Scheme of preparation of metallographic sections along with microhardness test pattern (1,2,3,4 and 5-number of the area where the measurement was taken).

The microhardness tests were conducted using the FISCHERSCOPE HM2000 microhardness tester (Helmut Fischer GmbH, Sindelfingen, Germany) with a test load of F = 0.05 N. The cross-sections of randomly selected specimens were embedded in resin, and the surfaces to be measured were prepared according to the methodology presented. To analyze local hardness values, measurements were carried out for 5 areas of the cross-section. For each area, 4 measurement points were determined, approximately 150 µm apart, as shown in Figure 3.

### 2.8. Uniaxial Tensile Tests

Uniaxial tensile tests were carried out on a Zwick Z400E testing machine with a macroXtens extensometer (ZwickRoell GmbH, Ulm, Germany) at ambient temperature in accordance with PN-EN ISO 6892-1:2020-05 [37]. The tests were performed for five specimens in each series. The values of tensile strength (R_m_), yield strength (R_p0.2_), and elastic modulus (E) were determined from stress–strain curves using the testXpert III v1.4 software (ZwickRoell GmbH, Ulm, Germany).

## 3. Results and Discussion

### 3.1. Relative Density and Structural Defects

The results of relative density measurements of samples made from Ti-6Al-4V and Ti-6Al-7Nb powders are shown in Table 3. For the Ti-6Al-4V alloy, the highest relative density value of 4.360 g/cm^3^ (98.42%) was obtained, and for the Ti-6Al-7Nb alloy the value was 4.485 g/cm^3^ (99.44%). In both cases, the highest relative density was observed for manufacturing strategy S8 (P = 250 W, v = 1200 mm/s, E = 70 J/mm^3^).

**Figure 4:**
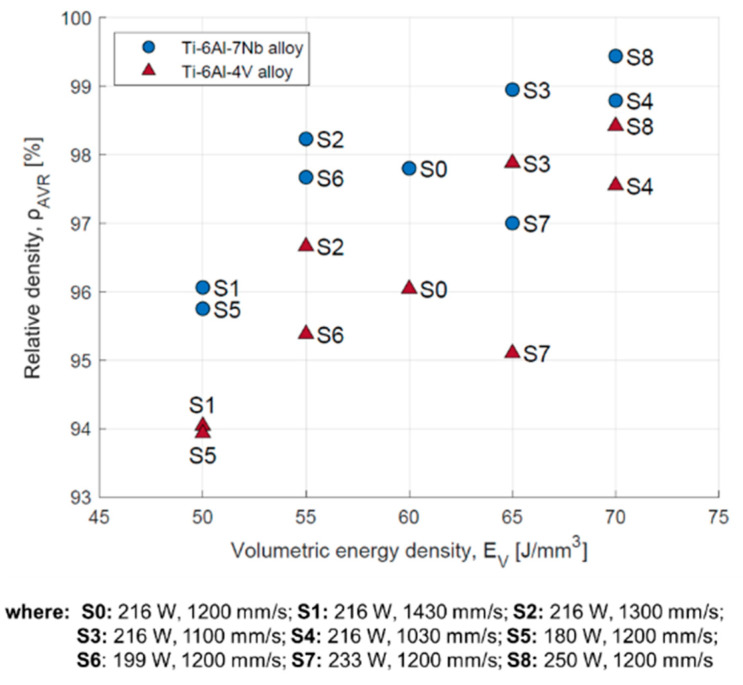
Relative density dependent on volumetric energy density for Ti-6Al-4V and Ti-6Al-7Nb alloys.

**Figure 5:**
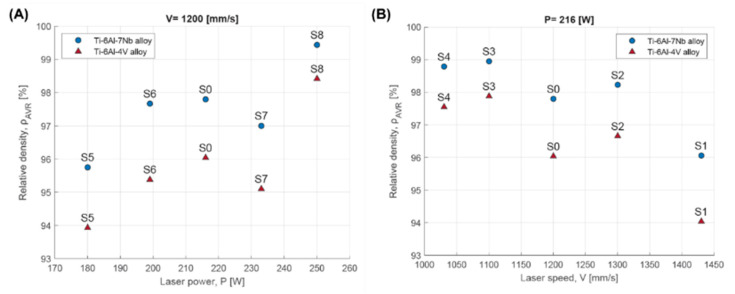
Relative density dependent on: (**A**) laser power; (**B**) scanning speed for Ti-6Al-4V and Ti-6Al-7Nb alloys.

It was found that it was possible to increase the relative density of the material by increasing the volumetric energy density (Figure 4). However, for the same laser energy densities, but for different combinations of scanning speed and laser power, different material relative density values were obtained. An analysis of the effect of selected parameters shows that increasing the laser power in combination with a constant scan speed increases the relative density of the material (Figure 5A). Increasing the scan speed while keeping the laser power constant decreases the relative density of the material (Figure 5B).

**Figure 6:**
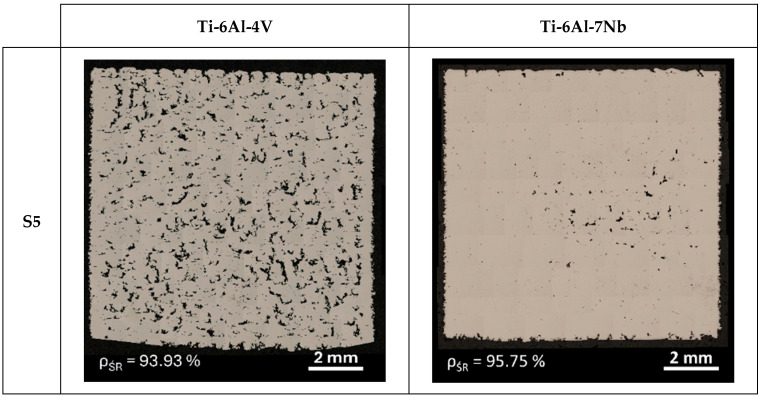
Mosaics of vertical cross-section of Ti-6Al-4V and Ti-6Al-7Nb samples with the lowest (S5) value of relative density.

**Figure 7:**
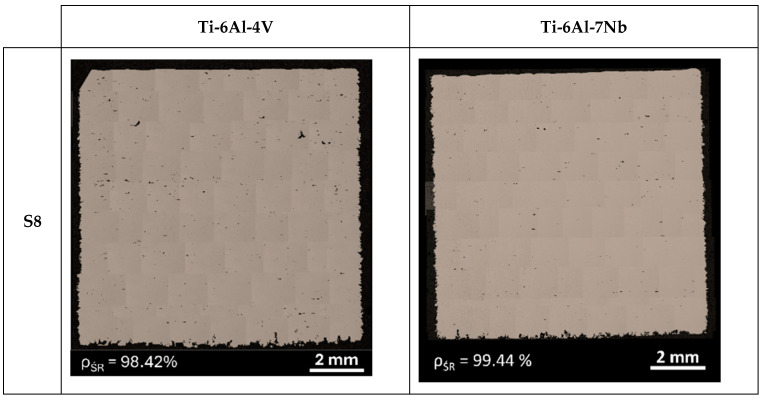
Mosaics of vertical cross-section of Ti-6Al-4V and Ti-6Al-7Nb samples with the highest (S8) value of relative density.

The porosity of a material’s internal structure is a determinant of the relative density. Figure 6 and Figure 7 show a mosaic of metallographic images for the vertical cross-section of samples with the lowest (S5) and highest (S8) relative densities for the Ti-6Al-4V and Ti-6Al-7Nb alloys. In the samples with the lowest relative density, pores with irregular and elongated shapes were observed, which can be classified as “lack-of-fusion” defects. The pores are arranged according to the direction of layer formation, and partially fused powder grains can be observed in some of them. 

The cause of this type of defects is, among other things, a volumetric energy density that is too low during the process. When the process is conducted with insufficient energy density, poor penetration of the molten metal pool occurs, leading to the incomplete melting of the material. Some unmelted powder grains may become trapped inside the pores. Bustillos et al. [42] demonstrated that a high scanning speed combined with insufficient laser power promotes the formation of “lack-of-fusion” defects. The studies by Liverani et al. [43] for 316 L stainless steel and by Aboulkhair et al. [44] on the AlSi10Mg alloy show that this relationship is an inherent characteristic of the SLM technology, independent of the construction material.

### 3.2. Surface

**Figure 8:**
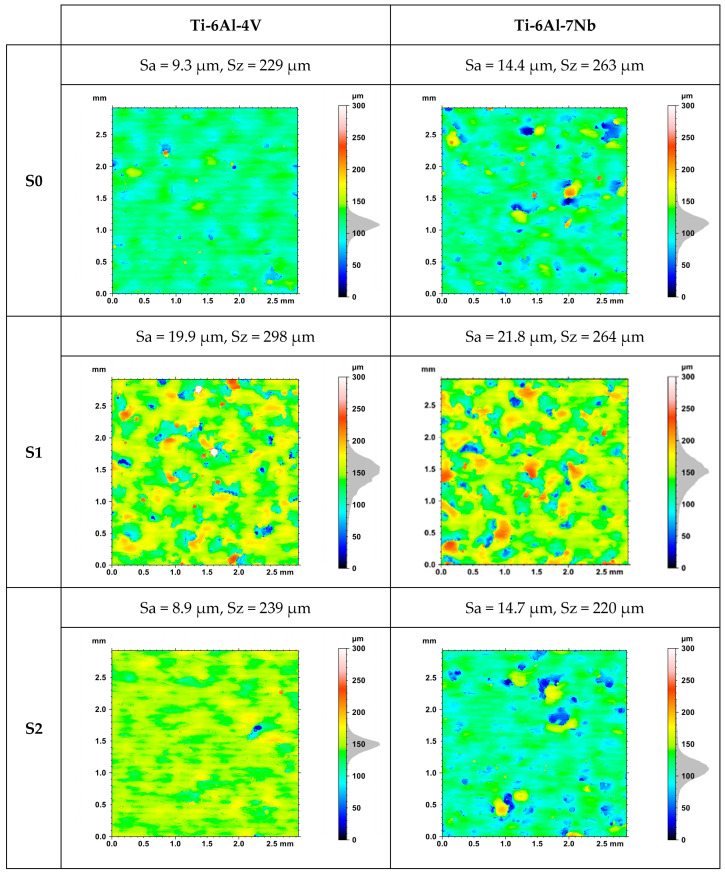

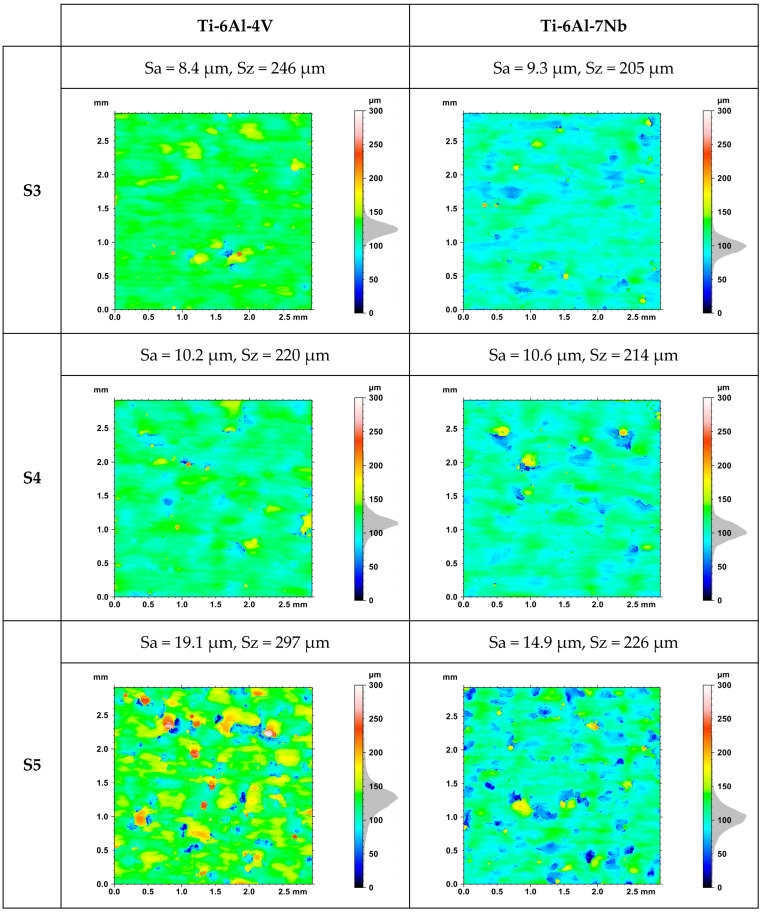

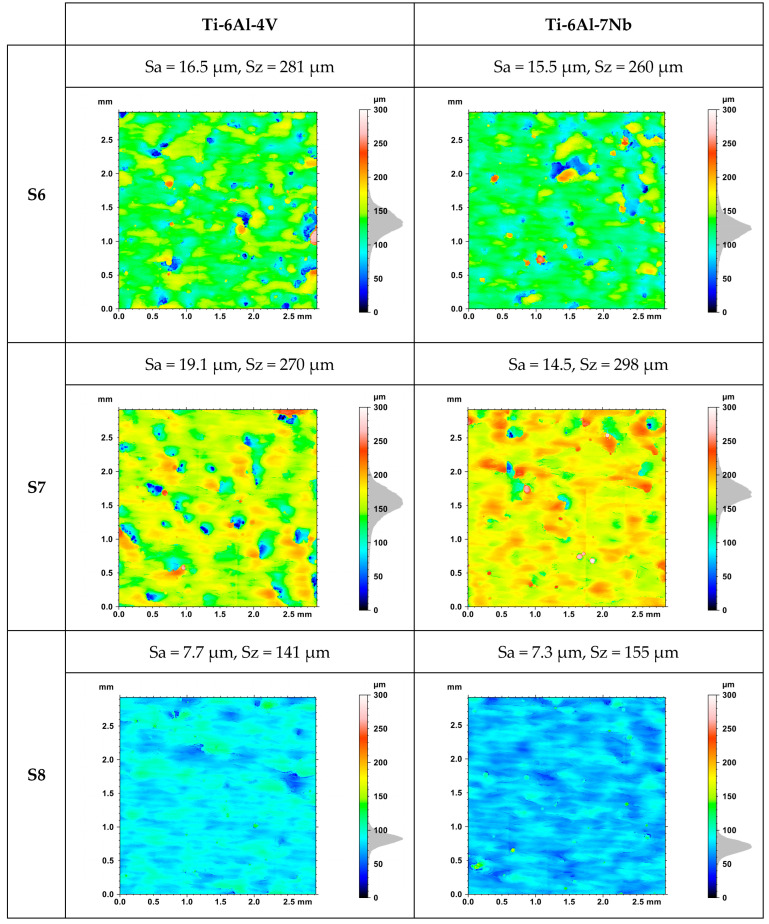
Topography of the top surface of randomly selected Ti-6Al-4V and Ti-6Al-7Nb samples.

Figure 8 presents images of the topography of the upper surface of a sample randomly selected from each series for Ti-6Al-4V and Ti-6Al-7Nb alloys. Differences in roughness parameter values were observed for samples produced using the same manufacturing strategy, which is related to the variable orientation of the position. This is further evidence of the importance of considering the spatial orientation of individual planes in the context of their surface quality.

**Figure 9:**
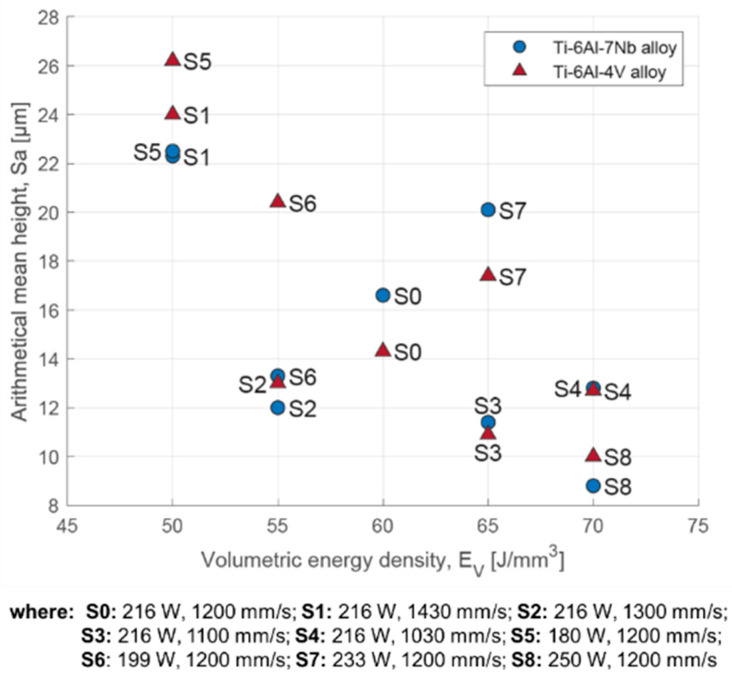
Surface roughness defined by the parameter Sa as a function of volumetric energy density for the Ti-6Al-4V and Ti-6Al-7Nb alloys.

Table 4 shows the evaluation of surface roughness based on the values of Sa and Sz parameters (averages of three measurements). A reduction in surface roughness was obtained as the results of an increase in the value of volumetric energy density (Figure 9). The lowest average value of the Sa was observed for strategy S8. This was, respectively, 10.0 ± 3.6 μm for the Ti-6Al-4V alloy and 8.8 ± 2.0 μm for the Ti-6Al-7Nb alloy.

**Figure 10:**
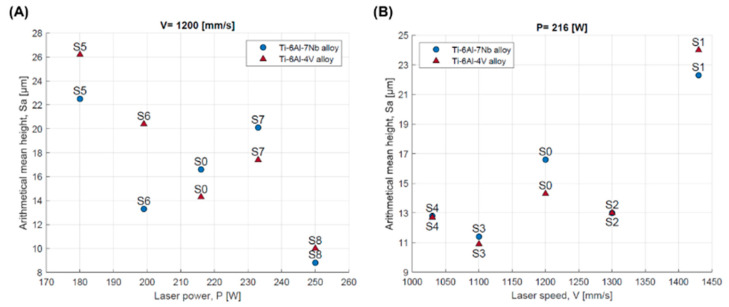
Surface roughness defined by the parameter Sa depending on: (**A**) laser power; (**B**) scanning speed for the Ti-6Al-4V and Ti-6Al-7Nb alloys.

The study showed a correlation of roughness with the values of laser power and scanning speed. A decrease in roughness is achieved by increasing the laser power without changing the scanning speed (Figure 10A), while an increase in roughness occurs when the laser power is held constant and the scanning speed is increased (Figure 10B).

**Figure 11:**
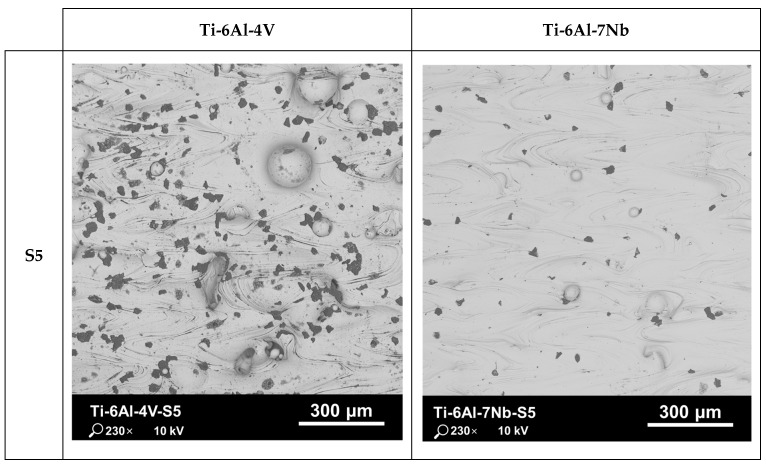

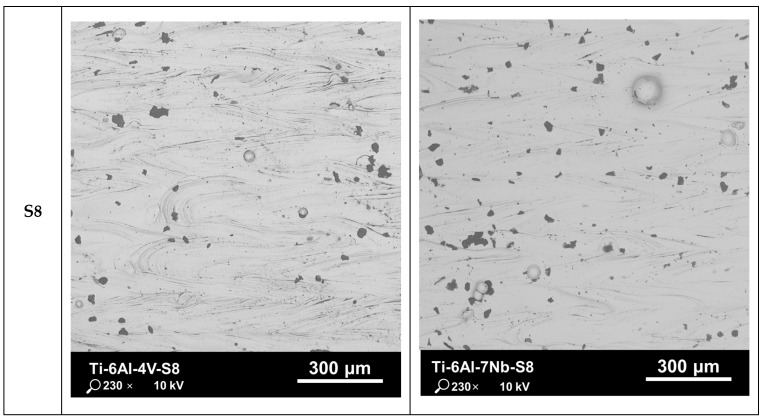
SEM morphology of the top surface of the Ti-6Al-4V and Ti-6Al-7Nb samples with the highest (S5) and the lowest (S8) value of surface roughness.

Figure 11 shows the SEM images of the top surfaces of samples with the highest (S5) and lowest (S8) surface roughness for the Ti-6Al-4V and Ti-6Al-7Nb alloys. 

The moving heat source (laser) creates unique marks on the top surface, known as laser welds. The welds exhibit characteristic ripples, which are the result of Marangoni convection [45]. Defects typical of SLM-manufactured surfaces were observed [46], including defects related to the so-called balling effect and the lack of complete melting of the powder. The occurrence of such defects may be related to the spatter effect and thermal diffusion. The movement of unmelted powder grains is caused by forces resulting from the surface tension gradient of the molten material. The high temperature of the melt pool causes the grains to partially melt, and when they cool, they settle at the edge of the pool [35,45]. The defects associated with the balling effect are larger and irregular in shape compared to agglomerates of unmelted or partially melted powder grains. The presence of the described defects depends on the process parameters, and their incidence increases with increasing volumetric energy density.

Increasing the surface roughness also affects the value of the determined relative density of the manufactured parts. Surface defects are not limited only to the top surface; they can also be observed on intermediate layers. Defects on the solidified surface of the intermediate layer can affect the distribution and thickness of the new powder layer. In such a situation, the delivered laser energy density may be insufficient to fully melt the material. Consequently, structural defects of the “lack-of-fusion” type may occur or be enlarged [19,27].

### 3.3. Microhardness

The results of the microhardness measurements of different areas of randomly selected samples made of the Ti-6Al-4V and Ti-6Al-7Nb alloy are shown in Table 5 (which occurred according to the scheme presented in Figure 3).

Due to the rapid heating, melting and cooling, the various fragments of components manufactured using SLM technology are subjected to multiple heating and cooling cycles, which affects their local structure and properties [36]. This helps in differentiating the microhardness of individual areas. It was observed that areas near the edges of the section (OB1–OB4) show higher hardness than the core area (OB5). For two-phase titanium alloys, including Ti-6Al-4V and Ti-6Al-7Nb, the hardness depends on the volume ratio of the β phase to the α (martensitic) phase [47]. On this basis, it can be assumed that there is segregation and concentration of the α phase at the edges of the samples. 

**Figure 12:**
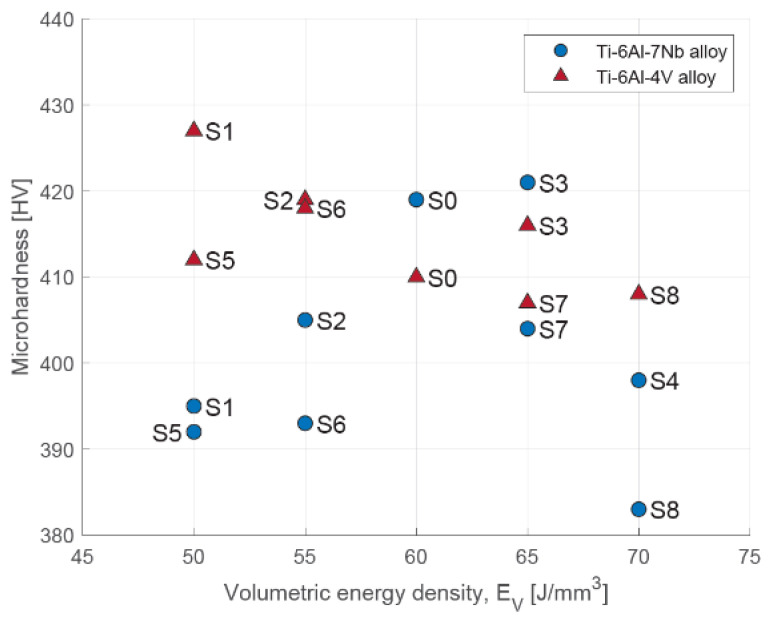
Microhardness of core area depending on volumetric energy density for Ti-6Al-4V and Ti-6Al-7Nb alloys.

The data presented in Table 5 are also presented graphically (Figure 12). In the case of the Ti-6Al-4V alloy, increasing the volumetric energy density resulted in a decrease in the hardness of the core region from 407 HV to 433 HV. Similar conclusions can be found in the work of Zhao et al. [36], although the values they obtained were lower, ranging from 371 HV to 384 HV. In the case of the Ti-6Al-7Nb alloy, the change in volumetric energy density initially resulted in an increase in the hardness of the core region; a significant decrease occurred after the exceedance of 65 J/mm^3^. The average core microhardness of Ti-6Al-7Nb ranged from 383 HV to 421 HV. These values are similar to those obtained by Chlebus et al. [32] (357 ± 18 HV) and Xu et al. [33] (371 ± 8 HV).

**Figure 13:**
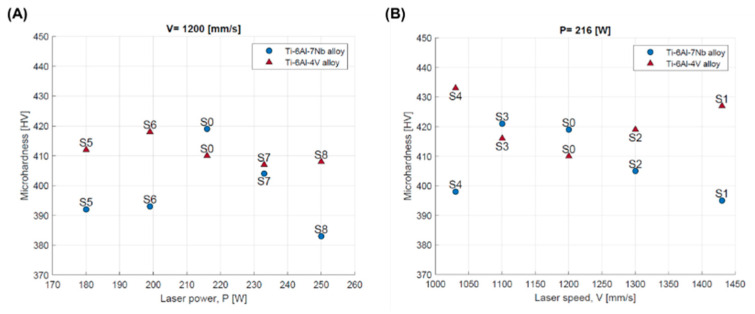
Microhardness of core area depending on: (**A**) laser power; (**B**) scanning speed for Ti-6Al-4V and Ti-6Al-7Nb alloys.

The change in scanning speed or laser power resulted in a change in the thermodynamic conditions within the melting pool. For the Ti-6Al-4V alloy, increasing the laser power did not lead to significant changes in microhardness—the measurement results for the sample produced at laser powers of 180 W (S5) and 250 W (S8) were 412 HV and 408 HV, respectively (Figure 13A). Increasing the scanning speed initially caused a decrease in the microhardness of the core area. Beyond 1200 mm/s, an increase in value was observed (Figure 13B).

In the case of the Ti-6Al-7Nb alloy, an increase in laser power initially results in an increase in microhardness in the core area, with a clear maximum for the S0 strategy. Beyond 216 W, a decrease in value was observed (Figure 13A). A similar dependency was observed with increasing scanning speed, with a maximum for strategy S3. Beyond 1100 mm/s, a decrease in the microhardness of the core area was observed (Figure 13B). 

Based on this, it can be assumed that the observed decrease in the microhardness of the core area was associated with an increased crystallization of the β phase in this area. The results may suggest that although both investigated alloys belong to two-phase alloys, they react differently to changes in the thermodynamic conditions prevailing in the pool of molten material caused by changes in laser power (temperature change) or scanning speed (change in heating and solidification time). 

### 3.4. Microstructure

**Figure 14:**
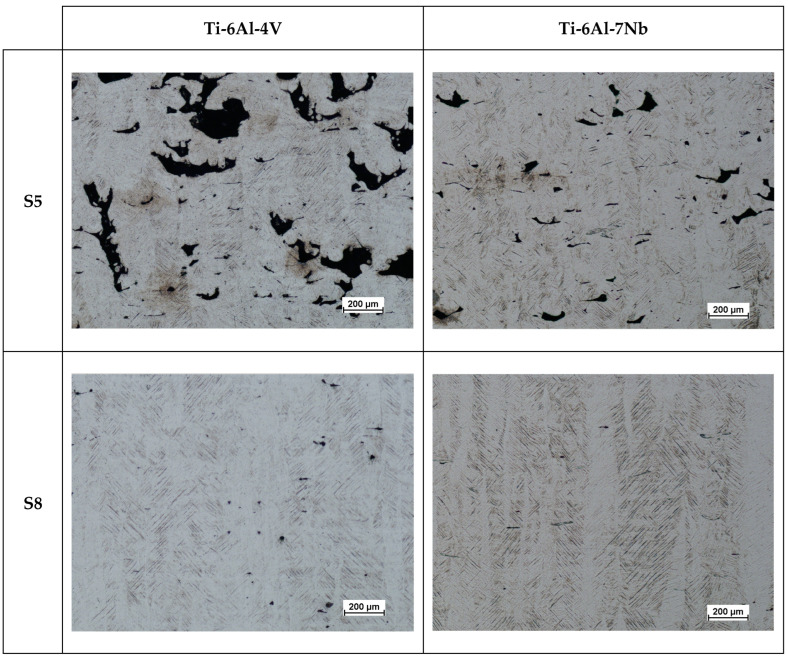
Microstructure of the Ti-6Al-4V and Ti-6Al-7Nb samples.

The as-built microstructures of the Ti-6Al-4V and Ti-6Al-7Nb alloys are shown in Figure 14. Regardless of the manufacturing strategy, the observed metallographic structure is typical for two-phase titanium alloys produced using SLM technology [48,49]. After etching, the columnar grains of the β phase, with their growth direction parallel to the build direction, were observed. The β phase crystallizes in a body-centered cubic (BCC) and is described as a solid solution of stabilizing elements, primarily V or Nb in this case [47]. The specificity of the SLM process, particularly the high solidification rates of the melting pool, promotes the crystallization of a metastable α’ phase within the β phase grains. The α’ phase crystallizes in a hexagonal close-packed (HCP) arrangement, and its microstructure is characterized by needle-like or plate-like features [47]. As demonstrated by the micrographs, the growth plane of the crystalline needles or plates of the α’ phase is oriented at an angle of approximately 45° to the growth of the β phase grains. Given that the generation of each successive layer requires the interaction of the laser beam with a layer that has already been solidified, the occurrence of a phase transformation from α” to β in this layer should be taken into account, as suggested by, among others, Chlebus et al. [32].

**Figure 15:**
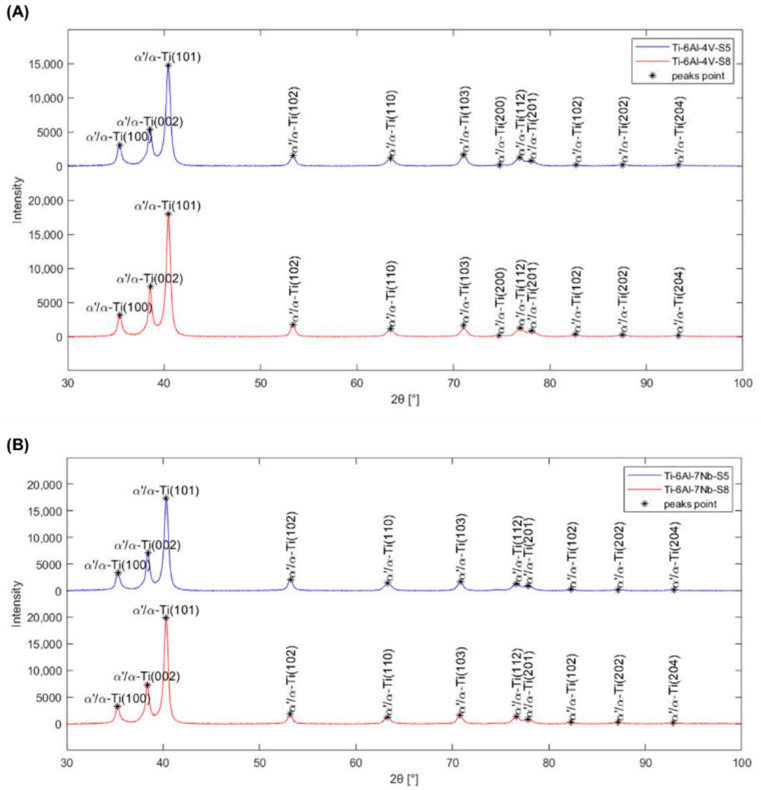
X-ray diffraction spectrums of (**A**) Ti-6Al-4V and (**B**) Ti-6Al-7Nb alloys manufactured according to strategies S5 and S8.

For a complete phase composition identification, X-ray diffraction analysis was conducted on samples cross-section for the Ti-6Al-4V and Ti-6Al-7Nb alloys manufactured according to strategies S5 and S8. Based on the fitting of diffraction patterns (Figure 15), it was found that for all tested alloys, the visible peaks are characteristic of the presence of titanium phase with a hexagonal close-packed (HCP) structure, due to the similarity of crystal lattice parameters [32,33,50].

In the obtained diffraction spectrums, no additional peaks were observed. Therefore, the presence of alloying additives and thermal processing conditions did not ensure the stabilization of the β-Ti phase. The Ti-6Al-4V and Ti-6Al-7Nb alloys manufactured using SLM technology exhibit non-equilibrium, brittle microstructures with metastable α’-phase martensite. This is consistent with findings in the literature [32,33,50].

### 3.5. Tensile Test Results

**Figure 16:**
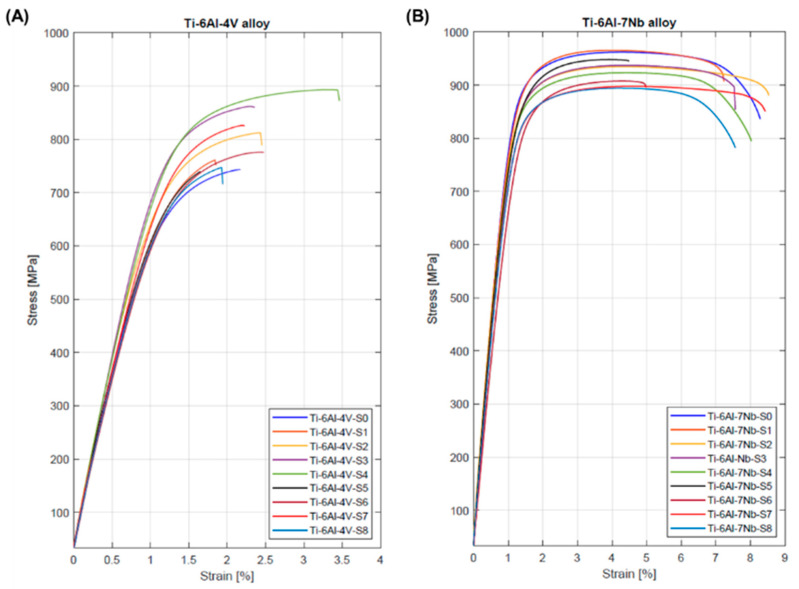
Stress–strain curve of (**A**) Ti-6Al-4V and (**B**) Ti-6Al-7Nb samples.

The similarity in the stress–strain curves for samples made using the same manufacturing strategy indicates a high repeatability of the SLM process. Therefore, Figure 16 depicts the stress–strain curves of randomly selected samples from each series made from the Ti-6Al-4V and Ti-6Al-7Nb alloys. The shape of the curves is typical for materials without a distinct yield point.

Table 6 presents a comparison of the values (an average of five measurements) of the elastic modulus (E), yield strength (R_p0.2_), tensile strength (R_m_), and elongation (A_25mm_) of the Ti-6Al-4V and Ti-6Al-7Nb alloys. 

In the case of the Ti-6Al-4V alloy, a clear increase in the values of all analyzed strength parameters was observed with an increase in volumetric energy density. A similar relationship was observed by Zhao et al. [36]. The tensile properties of the Ti-6Al-4V alloy observed in this study are lower in comparison to the range reported in previous research [36,51,52,53]. 

Conversely it was observed that, for the Ti-6Al-7Nb alloy, the tensile properties decreased with the increase in volumetric energy density. Due to the small number of literature reports, it is difficult to verify this observation. Chlebus et al. [32] reported that the tensile strength of samples from Ti-6Al-7Nb alloy with a vertical direction of layer building in relation to the working platform was 776 ± 40 MPa (the values of the other parameters are unavailable). This value is approximately 13–25% lower than that reported in this work. Unfortunately, Xu et al. p [33] did not provide clear information about the direction of layer building in the samples they analyzed, which, as is known, has a significant impact on the mechanical properties of alloys produced by SLM technology (anisotropy). However, the yield strength and tensile strength values obtained by Xu et al. were 1082 ± 13 MPa and 1160 ± 18 MPa, respectively. They are approximately 23–34% and 17–25% lower than reported in this work.

The Ti-6Al-4V alloy achieved lower values of the analyzed tensile properties parameters compared to the Ti-6Al-7Nb alloy. This is particularly evident in the case of elongation. For the Ti-6Al-7Nb alloy, the highest elongation value was 7.6 ± 0.9% and was approximately four times higher than the highest elongation for the Ti-6Al-4V alloy (2.2 ± 0.2%).

**Figure 17:**
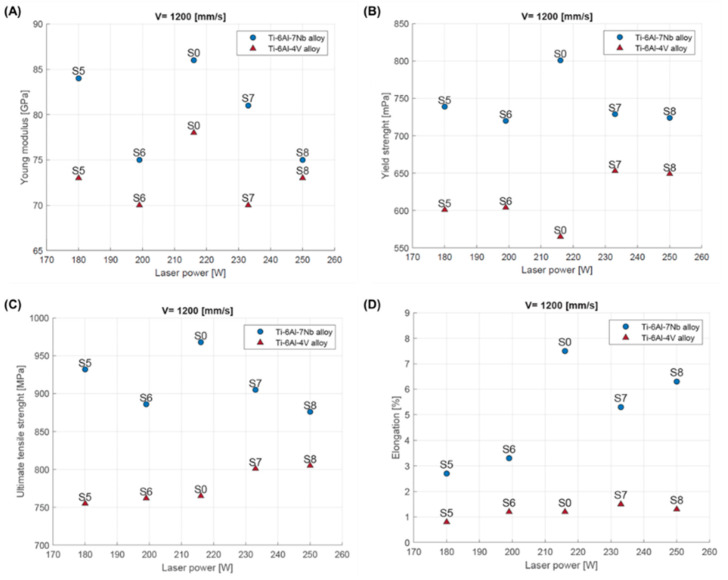
Mechanical properties of Ti-6Al-4V and Ti-6Al-7Nb depending on laser power: (**A**) Young’s modulus; (**B**) yield strength; (**C**) tensile strength (Rm); (**D**) elongation.

The study showed that changing the process parameters had a significant effect (laser power and scanning speed) on the analyzed mechanical properties of both alloys. In general, increasing the laser power while maintaining a constant scanning speed led to an increase in the yield strength, tensile strength, and elongation for the Ti-6Al-4V alloy. Conversely, for the Ti-6Al-7Nb alloy, a decrease in the Young’s modulus, yield strength, and tensile strength values was observed, alongside an increase in the elongation value (Figure 17).

## 4. Conclusions

The aim of the study was to determine the effect of varying the values of the process parameters (scanning speed and laser power) on the relative density, microstructure and mechanical properties of the Ti-6Al-4V and Ti-6Al-7Nb alloys. A comparison was made between the processability and properties of the two alloys produced using the same process strategies. These studies are particularly important for the Ti-6Al-Nb alloy, which is becoming increasingly popular in medicine. The currently available number of publications definitely does not exhaust the needs of the additive manufacturing industry.

For both tested materials, considering the maximization of relative density and minimization of surface roughness as selection criteria, the best strategy was S8, where the laser energy density was 70 J/mm3 with a laser power of 250 W and a scanning speed of 1200 mm/s. Regardless of the structural material used, the relative density and surface roughness can be controlled by changing the laser power or scanning speed. However, it is more advantageous to increase the laser power in order to increase the relative density while minimizing the surface roughness by adjusting the volumetric energy density.

The microstructures of the two investigated alloys were similar when they were manufactured under similar conditions. In both cases, a typical microstructure for titanium alloys manufactured by SLM technology was obtained, consisting of columnar β phase grains with a needle-like α’ phase inside.

For all manufacturing strategies tested, higher relative density values were obtained for the Ti-6Al-7Nb alloy. In addition, increasing the volumetric energy density increased the tensile strength of the Ti-6Al-4V alloy, while the opposite relationship was observed for the Ti-6Al-7Nb alloy. The investigated Ti-6Al-7Nb alloy exhibited higher elongation values. The core microhardness of the Ti-6Al-7Nb alloy samples was lower than that of the Ti-6Al-4V alloy. The obtained microhardness and elongation results suggest that the Ti-6Al-7Nb alloy solidified with a higher volume of β phase.

The results show that these changes in the parameters of the fabrication process result in different material properties for the Ti-6Al-4V and Ti-6Al-7Nb alloys. The selected criteria for choosing a fabrication strategy are both practical and economical. Increased relative density in the raw state translates into better mechanical properties. Conversely, reduced roughness leads to lower time and financial costs for finishing operations. It should be noted that improvements in the microhardness and mechanical properties, including elongation, of titanium-based alloys produced by SLM technology can be achieved by appropriate heat treatment, which is a future direction of the work of the authors of this publication.

## Figures and Tables

**Table 1:** Chemical compositions of Ti-6Al-4V and Ti-6Al-7Nb powders (wt. %).

Powder	Ti	Al	V	Nb	Fe	O	C	N	H
**Ti-6Al-4V**	Balance	6.00	4.00	-	≤0.25	≤0.13	≤0.08	≤0.03	≤0.012
**Ti-6Al-7Nb**	Balance	6.05	-	7.1	0.15	0.08	0.015	0.016	0.001

**Table 2:** Manufacturing strategies for Ti-6Al-4V and Ti-6Al-7Nb.

Manufacturing Strategy Symbol	Parameters				
	P [W]	v [mm/s]	h [mm]	t [mm]	E_v_ [J/mm^3^]
S0	216	1200	0.1	0.03	60
S1		1430			50
S2		1300			55
S3		1100			65
S4		1030			70
S5	180	1200			50
S6	199				55
S7	233				65
S8	250				70

**Table 3:** Relative density of Ti-6Al-4V and Ti-6Al- 7Nb fabricated by SLM.

Manufacturing Strategy Symbol	Ti-6Al-4V		Ti-6Al-7Nb	
	ρ_AVR_ [g/cm^3^]	ρ_AVR_ [%]	ρ_AVR_ [g/cm^3^]	ρ_AVR_ [%]
**S0**	4.254 ± 0.03	96.04	4.411 ± 0.03	97.80
**S1**	4.166 ± 0.01	94.04	4.332 ± 0.03	96.06
**S2**	4.282 ± 0.04	96.66	4.430 ± 0.02	98.23
**S3**	4.336 ± 0.02	97.88	4.462 ± 0.02	98.95
**S4**	4.322 ± 0.03	97.55	4.456 ± 0.02	98.79
**S5**	4.161 ± 0.06	93.93	4.318 ± 0.05	95.75
**S6**	4.225 ± 0.02	95.38	4.405 ± 0.02	97.67
**S7**	4.213 ± 0.03	95.10	4.375 ± 0.02	97.00
**S8**	4.360 ± 0.01	98.42	4.485 ± 0.01	99.44

where the values, as 100% of the material density, are 4.43 g/cm^3^ for Ti-6Al-4V alloy and 4.51 g/cm^3^ for Ti-6Al-7Nb alloy.

**Table 4:** Sa and Sz values for the upper surfaces of Ti-6Al-4V and Ti-6Al-7Nb samples.

Manufacturing Strategy Symbol	Ti-6Al-4V		Ti-6Al-7Nb	
	Sa [μm]	Sz [μm]	Sa [μm]	Sz [μm]
**S0**	14.3 ± 4.5	267 ± 50	16.6 ± 2.3	290 ± 25
**S1**	24.0 ± 3.3	350 ± 13	22.3 ± 4.4	282 ± 28
**S2**	13.0 ± 4.9	303 ± 61	13.0 ± 1.6	197 ± 22
**S3**	10.9 ± 3.2	244 ± 36	11.4 ± 2.2	209 ± 7
**S4**	12.7 ± 1.8	244 ±19	12.8 ± 2.2	238 ± 18
**S5**	26.2 ± 5.2	311 ± 52	22.5 ± 5.4	306 ± 78
**S6**	20.4 ± 5.2	315 ± 24	13.3 ± 1.5	253 ± 16
**S7**	17.4 ± 2.0	256 ± 32	20.1 ± 4.1	356 ± 41
**S8**	10.0 ± 3.6	184 ± 45	8.8 ± 2.0	187 ± 31

**Table 5:** Microhardness of Ti-6Al-4V and Ti-6Al-7Nb samples.

Manufacturing Strategy Symbol	HV_0.05_				
	OB1	OB2	OB3	OB4	OB5
**Ti-6Al-4V**					
**S0**	438 ± 11	426 ± 7	449 ± 4	415 ± 10	410 ± 14
**S1**	423 ± 8	414 ± 6	454 ± 17	418 ± 36	427 ± 8
**S2**	446 ± 16	424 ± 10	420 ± 5	416 ± 30	419 ± 5
**S3**	436 ± 4	419 ± 9	441 ± 13	436 ± 8	416 ± 4
**S4**	427 ± 20	418 ± 13	429 ± 9	438 ± 4	433 ± 7
**S5**	422 ± 10	420 ± 5	421 ± 9	411 ± 9	412 ± 13
**S6**	421 ± 3	424 ± 11	413 ± 8	405 ± 12	418 ± 6
**S7**	435 ± 6	414 ± 13	428 ± 13	426 ± 8	407 ± 26
**S8**	420 ± 6	411 ± 12	413 ± 18	408 ± 25	408 ± 5
**Ti-6Al-7Nb**					
**S0**	420 ± 7	423 ± 11	428 ± 8	448 ± 6	419 ± 5
**S1**	402 ± 7	406 ± 10	430 ± 35	436 ± 4	395 ± 5
**S2**	418 ± 7	433 ± 14	406 ± 12	430 ± 5	405 ±4
**S3**	431 ± 7	420 ± 32	435 ± 9	433 ±8	421 ± 14
**S4**	442 ± 5	436 ± 11	422 ± 10	435 ± 13	398 ± 3
**S5**	424 ± 12	416 ± 10	428 ± 8	423 ± 8	392 ± 11
**S6**	416 ± 9	410 ±5	349 ± 12	424 ± 8	393 ± 6
**S7**	407 ± 10	435 ± 8	390 ± 10	432 ± 2	404 ± 6
**S8**	394 ± 9	403 ± 12	386 ± 4	380 ± 11	383 ± 1

**Table 6:** Young’s modulus, yield strength, tensile strength and elongation of Ti-6Al-4V and Ti-6Al-7Nb samples.

Manufacturing Strategy Symbol	E [GPa]	R_p0.2_ [MPa]	R_m_ [MPa]	A_25mm_ [%]
**Ti-6Al-4V**				
**S0**	78 ± 12	565 ± 51	765 ± 22	1.2 ± 0.1
**S1**	67 ± 4	591 ± 22	724 ± 33	0.9 ± 0.2
**S2**	76 ± 4	588 ± 26	784 ± 25	1.4 ± 0.3
**S3**	80 ± 9	686 ± 54	860 ± 31	1.6 ± 0.5
**S4**	79 ± 5	726 ± 64	912 ± 17	2.2 ± 0.2
**S5**	73 ± 2	601 ± 20	755 ± 25	0.8 ± 0.1
**S6**	70 ± 4	604 ± 15	762 ± 11	1.2 ± 0.2
**S7**	70 ± 4	653 ± 19	801 ± 28	1.5 ± 0.3
**S8**	73 ± 4	649 ± 40	805 ± 38	1.3 ± 0.2
**Ti-6Al-7Nb**				
**S0**	86 ± 5	801 ± 13	968 ± 8	7.5 ± 1.0
**S1**	88 ± 3	789 ± 13	977 ± 17	5.4 ± 0.6
**S2**	84 ± 3	779 ± 18	945 ± 20	5.7 ± 2.4
**S3**	83 ± 3	769 ± 13	925 ± 16	7.6 ± 0.9
**S4**	82 ± 4	778 ± 13	932 ± 9	7.5 ± 0.6
**S5**	84 ± 2	739 ± 7	932 ± 8	2.7 ± 1.1
**S6**	75 ± 5	720 ± 25	886 ± 24	3.3 ± 0.9
**S7**	81 ± 4	729 ± 25	905 ± 15	5.3 ± 1.5
**S8**	75 ± 3	724 ± 28	876 ± 17	6.3 ±1.5

## Data Availability

The data presented in this study are available on request from the corresponding author.
